# Quantifying the Lagged Effects of Climate Variables on Malaria Risk in Eastern Uganda

**DOI:** 10.4269/ajtmh.25-0031

**Published:** 2025-10-07

**Authors:** Sooyoung Kim, Betty Nabukeera, Yehu Taremwa, Maureen Ng’etich, Flavian Otieno, Steve Cygu, Dan Kajungu, Agnes Kiragga, Yesim Tozan

**Affiliations:** ^1^ Department of Public Health Policy and Management, School of Global Public Health, New York University, New York, New York;; ^2^ Makerere University Center for Health and Population Research, Iganga-Mayuge Health and Demographic Site, Makerere University, Kampala, Uganda;; ^3^ Data Science Program, Research Division, African Population and Health Research Center, Nairobi, Kenya;; ^4^ Department of Global Health, Stellenbosch University, Stellenbosch, South Africa;; ^5^ Department of Global and Environmental Health, School of Global Public Health, New York University, New York, New York

## Abstract

Climate change is anticipated to significantly affect malaria transmission. Previous research has shown lagged, nonlinear associations between climate variables and malaria risk, with highly context-specific exposure-lag-response relationship. Using weekly malaria case data collected between July 2018 and February 2023 from a health facility within the Iganga-Mayuge Health and Demographic Surveillance System site in Uganda and remotely sensed temperature and rainfall data, we quantified the associations between temperature and rainfall and risk of developing symptomatic malaria using a distributed lag nonlinear model. Furthermore, we investigated whether these associations varied by age group. Our analysis revealed a lag of 2 to 8 weeks between exposure to rainfall exceeding 200 mm/week and a significant increase in the risk of developing symptomatic malaria; no statistically significant lagged association was found with temperature. Additionally, the risk in school-aged children was less sensitive to climate variables compared with the other age groups. Rainfall was found to be associated with an increased risk at a lag of 2 months at the study site. This finding provides valuable guidance for local health authorities in determining the optimal timing for preventive interventions and in preparing for the anticipated rise in demand for malaria case management. The observed variations in the risk of developing symptomatic malaria across different age groups highlight the need for targeted interventions tailored to specific populations. Overall, the significant associations between climate variables and malaria risk underscore the importance of context-specific, adaptive malaria control strategies, complemented by broader efforts to mitigate climate change.

## INTRODUCTION

The global burden of malaria has remained a persistent public health challenge, particularly in sub-Saharan Africa (SSA).^1^ The WHO estimated that there were approximately 249 million malaria cases and 608,000 deaths globally in 2022, with Africa accounting for over 90% of both cases and fatalities.^1^ Within SSA, malaria exerts a significant burden on vulnerable populations, particularly young children and pregnant women.^1^^,^^2^

Uganda, a landlocked country located in the heart of SSA along the equator, experiences high rates of malaria transmission. In 2022, Uganda reported the third largest increase in malaria incidence, with an additional 597,600 cases.^1^ Malaria is transmitted year-round in Uganda,^3^ with transmission predominantly concentrated in rural areas.^4^ Factors such as limited access to health care services, socioeconomic disparities, and climatic conditions that promote mosquito breeding contribute to the persisting burden of the disease.^3^^,^^5^^–^^7^ Improved housing and the use of insecticide-treated bed nets significantly lower the risk of malaria infection, whereas farming and other outdoor activities increase exposure to the disease.^8^^,^^9^ Malaria takes a heavy toll on young children, pregnant women, and communities residing near Lake Victoria, where year-round high temperatures and two rainy seasons create ideal conditions for transmission.^7^

Climate change is expected to significantly impact malaria transmission and prevalence globally.^10^^,^^11^ Changes in temperature, rainfall patterns, and humidity can affect the life cycle of malaria parasites, the behavior and population dynamics of malaria vectors, and the dynamics of human–mosquito interactions.^11^ Although warmer temperatures can shorten the incubation period of malaria parasites within mosquitoes, potentially increasing transmission rates^12^^,^^13^ in places where daily minimum temperatures fall below 20°C, the malaria parasites’ growth cycle within mosquitoes is interrupted, preventing transmission to human hosts.^14^ Moderate to heavy rainfall can either promote the proliferation of malaria vectors or disrupt mosquito breeding sites through flooding,^15^^,^^16^ affecting the density and distribution of malaria vectors.^17^^,^^18^ Additionally, contextual factors such as human behavior and socioeconomic conditions can further moderate the relationship between climate variables and malaria risk.

Adapting and strengthening malaria control strategies in the context of broader climate change mitigation and adaptation are critical to minimizing the projected impacts of climate change on malaria. This crucial need to adapt and strengthen malaria control strategies underscores the importance of understanding the complex associations between climate variables and malaria risk, which could inform the development of climate-informed interventions for malaria. Previous studies have documented lagged associations between climate factors—such as temperature, rainfall, and humidity—and malaria risk.^19^ In tropical countries, particularly in SSA (with the exception of South Africa), a 7- to 12-week lag between rising temperatures and malaria risk and an 8- to 12-week lag between increased rainfall and malaria risk have been observed.^19^ However, as mentioned, exposure-lag-response relationships are shaped by a range of factors, making them highly context-specific.

Using a distributed lag nonlinear model (DLNM), this study aimed to quantify the associations between climate variables and malaria cases in a longitudinal population cohort at the Iganga-Mayuge Health and Demographic Surveillance System (HDSS) site, located in a highly endemic area in Uganda. Given the age-specific vulnerability to malaria, we also examined whether these associations varied by age group using data on malaria cases for three subgroups: children under 5 years, school-aged children (age 5–14 years), and individuals aged 15 years or older. The findings from this study have the potential to inform the development of climate-driven and functional early-warning systems for malaria, enabling the timely deployment of targeted interventions for the age groups most at risk.

## MATERIALS AND METHODS

### Study setting.

The Iganga-Mayuge HDSS site has been serving as a community-based population cohort since its establishment in 2004. It is located across the Iganga and Mayuge districts in Eastern Uganda and is approximately 120 km east of the capital city, Kampala. The site covers 65 villages across seven subcounties over an area of 155 km^2^.^20^ In 2023, the population in the HDSS catchment area was 101,499 across 20,500 households, with an average annual growth rate of 4.8% and had a crude birth rate about five times higher than the crude death rate. The population is predominantly Muslim, and the HDSS site has a mix of rural (49%) and peri-urban (51%) communities.^20^ This HDSS site is located within the Lake Victoria climate zone,^21^^,^^22^ and the Busoga region within which the HDSS site is located has a high malaria incidence of 195 cases per 1,000 persons.^23^ Currently, there are 16 community health facilities and two hospitals in the area.^20^

### Malaria data.

In 2018, the HDSS site established an electronic health record and linkage system with a community health facility within its catchment area. Since then, this health facility has collected detailed information during each community member’s facility visit, which was then integrated with the demographic and socioeconomic information collected through the HDSS biannual surveys.^20^ In this study, we used anonymized patient data collected between July 2018 and February 2023 and aggregated daily reported malaria cases into weekly totals to create a time-series dataset of malaria cases. We assessed the completeness of the analytical dataset and identified days with no recorded malaria cases. To account for potential gaps in the time series, we generated a complete calendar spanning July 1, 2018 to February 28, 2023. We assumed that the absence of reported cases on a given day indicated zero incidence and assigned a value of zero to these dates to enable accurate weekly aggregation of malaria case counts.

### Climate data.

The weekly malaria case data were merged with the climate data from three distinct sources: Complete ERA5 global atmospheric reanalysis with a spatial resolution of 0.25° × 0.25°^24^ and ERA5-Land daily aggregated-European Centre for Medium-Range Weather Forecasts (EMCWF) climate reanalysis with a spatial resolution of 0.1° × 0.1°,^25^ both from Copernicus Climate Change Service (C3s) Climate Data Store (CDS) and MOD21A1D.061 Terra Land Surface Temperature and 3-Band Emissivity Daily Global^26^ from National Aeronautics and Space Administration (NASA) Land Processes Distributed Active Archive Center (LP DAAC) with a spatial resolution of 1 km × 1 km. We extracted weekly total rainfall (mm) and average temperature (°C) data from all three datasets using the HDSS site’s shapefile. Instead of using the exact polygon defined by the shapefile, we used the geocoordinates of the centroid and applied a 10-km buffer around it to better account for inaccuracies in the centroid coordinates and the limited resolution of the climate data. Specifically, the 10-km buffer was selected as a reasonable compromise that captures local variation in climate conditions without extending far beyond the area represented by the HDSS site. Additionally, we extracted weekly data on minimum and maximum temperatures (°C) from the ERA-Land daily aggregated-EMCWF climate reanalysis using the same methods. For the Complete ERA5 global atmospheric reanalysis data, data extraction was performed using the R statistical software with *raster*^27^ and *sf*^28^ packages, and Google Earth Engine^29^ was used for the other two datasets.

### Modeling approach.

First, we fitted a DLNM model^30^ and used a quasi-Poisson model because it is a more flexible model for time-series data than the Poisson model and better accommodates overdispersion in count data (e.g., malaria cases). The DLNM framework employs the concept of a cross-basis function to combine distributed lag models^31^ with nonlinear functions^31^ and allows us to analyze the delayed and nonlinear effects of environmental exposures (e.g., temperature, rainfall) on disease outcomes (e.g., malaria cases)^30^ over a range of lags (i.e., time periods). The simplest form of the DLNM model incorporating rainfall and temperature as the exposure variables and malaria cases as the outcome variable is expressed as follows:

$$M_{t}∼\text{quasiPoisson}\left(\mu_{t}\right)\;E\left(M_{t}\right)=\beta +f_{\text{rain}}\left({\text{Rain}}_{t},\;df_{\text{rain}},\;df_{\text{lagrain}}\right)+f_{temp}({\text{Temp}}_{t},\;df_{\text{temp}},\;df_{\text{lagtemp}})+s\left(T_{t},\;df_{\text{time}}\right)$$ (Equation 1)

In the above equation, $M_{t}$ represents the malaria case count in week *t* and follows a quasi-Poisson distribution with a mean number of malaria cases of $\mu_{t}$. $\mu_{t}$ can also be expressed as $E(M_{t})$, the expected number of malaria cases in week *t*. $f({\text{Var}}_{t}\;df_{\text{var}},\;df_{\text{lag}})$ is the cross-basis function for temperature and rainfall with a corresponding spline function *f* and degrees of freedom ($df_{\text{var}}$) and a lag-response relationship ($df_{\text{lag}})$. $s(T_{t},\;df_{\text{time}})$ is a smoothed time function with a corresponding spline function *s* and degrees of freedom ($df_{\text{time}}$).

### Model building and selection.

Model building and selection in DLNM present a significant challenge due to the wide range of possible choices for model elements.^30^ This process entails testing of various model parameters, including different types of spline functions, placement of spline knots, degrees of freedom, and lag dimensions. In this study, we explored climate data from three different sources for a number of climate variables and experimented with a diversity of shapes for the cross-basis and smoothed time functions, defined by spline functions and lag structures, to identify the best-performing model. To evaluate model performance, we used both the Akaike information criterion (AIC) and the Bayes information criterion (BIC).^32^ For both criteria, lower values indicate better model fit. Although prior studies often relied on the BIC due to its stronger penalization of models with a large number of parameters—favoring more parsimonious models over complex ones—the AIC is generally recommended for DLNM.^30^ Therefore, when the two criteria conflicted, we prioritized the AIC over the BIC in selecting the final model. Supplemental Table 1 provides a summary of the various model aspects evaluated to determine the final model.

Specifically, we first tested various spline functions with different knot placements and degrees of freedom to identify the best-performing DLNM model, as outlined in Equation 1 above. This process was repeated for each climate dataset, allowing us to choose the dataset that performed best based on the AIC/BIC criteria. Using the chosen dataset, we then applied a stepwise model-building approach to test different combinations of model parameters, ultimately arriving at the best-performing model.

### Age group–specific analysis.

Malaria epidemiology in SSA displays age-specific patterns of susceptibility and exposure.^2^^,^^3^^,^^5^^,^^6^^,^^33^^,^^34^ Children under 5 years of age bear a disproportionate burden of severe malaria, accounting for over two-thirds of all malaria-related deaths.^33^^,^^35^ Key risk factors include their weaker immune systems, limited access to malaria prevention and treatment, and household-level socioeconomic vulnerabilities.^5^^,^^33^ Conversely, school-aged children in SSA have the highest prevalence of malaria, with over 70% having parasitemia in highly endemic areas.^34^ For this reason, school-aged children have often been a key target for malaria control interventions.^36^ To conduct a more nuanced analysis, we investigated whether the associations between climate variables and malaria cases varied by age group. Specifically, we aggregated weekly malaria case data for three distinct age groups: children under 5 years of age, school-aged children (5–14 years), and individuals aged 15 years or older. Using this age-specific data, we applied the best performing model from the previous step to examine the exposure-lag-response relationships between climate variables and malaria cases across these different age groups.

### Data availability statement.

All analysis were conducted in R statistical software using the package *dlnm.*^37^ All the data and R codes that support the findings of this study are available in the GitHub repository (https://github.com/sk9076/Uganda_HDSS).

## RESULTS

### Descriptive analysis.

**Figure 1.**
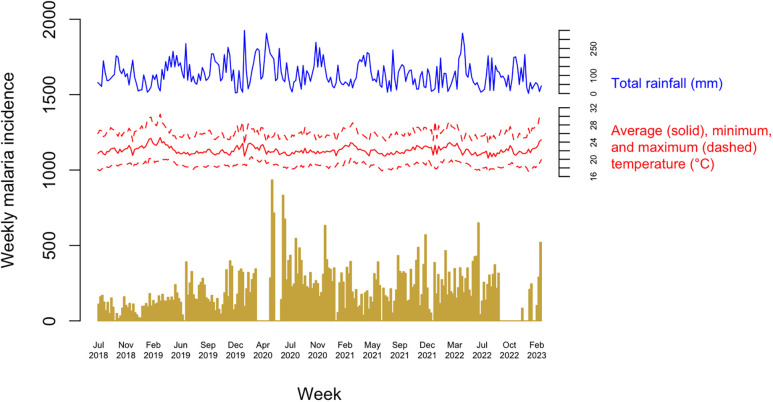
Weekly facility-based reported malaria cases and climate variables (rainfall and temperature based on ERA5 daily climate data) at the Iganga-Mayuge Health and Demographic Surveillance System (HDSS) site, Uganda, July 2018–February 2023.

**Figure 2.**
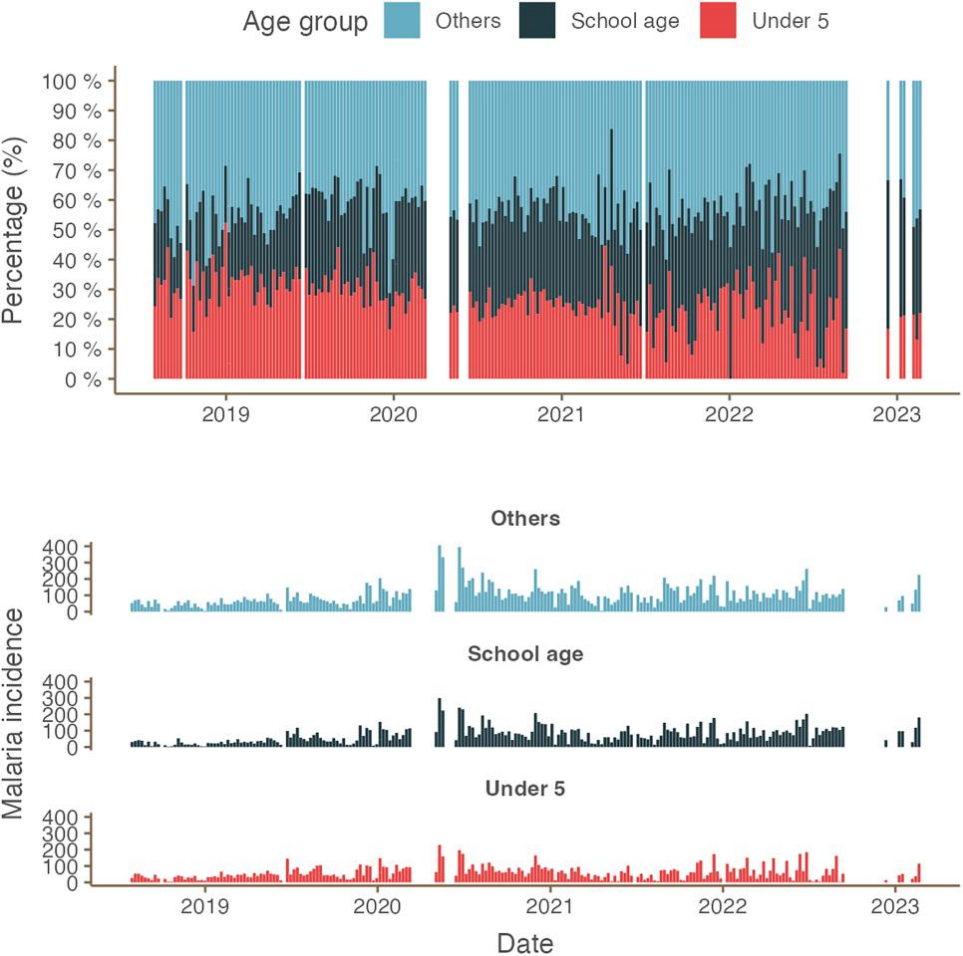
Weekly reported malaria cases by age group at the Iganga-Mayuge Health and Demographic Surveillance System (HDSS) site, Uganda, July 2018–February 2023.

Table 1 provides a summary of the descriptive analysis results for the climate variables using the ERA5 daily climate data. Figure 1 shows the weekly reported malaria cases between July 2018 and February 2021 from a single facility in the Iganga-Mayuge HDSS site, along with the ERA5 daily climate data. During this period, a total of 48,311 malaria cases were recorded, displaying seasonal fluctuations with 2 peaks each year, one in the months May–July and the other in in the months November–January. The number of cases significantly increased in 2020, followed by a gradual decline in subsequent years, although the overall number remained higher than the years preceding 2020. Weekly mean daily temperatures ranged from 20.3°C to 25.1°C, with the hottest days occurring in February/March and the coolest in July/August. Total weekly rainfall ranged between 0.65 mm and 349.3 mm, with higher rainfall observed during April/May (Supplemental Figure 1). The ERA5 hourly dataset showed the same pattern (Supplemental Figure 2) as the ERA5 daily data. The Moderate Resolution Imaging Spectroradiometer (MODIS) Terra Land Surface Temperature dataset was deemed unsuitable for building DLNM models due to data sparseness (Supplemental Figure 3) and was excluded from further analysis. The temporal pattern of reported malaria cases was consistent across age groups (Figure 2).

**Table 1:** Descriptive analysis summary

Variable	Minimum	1st quartile	Median	Mean	3rd quartile	Maximum
Weekly total rainfall (mm)	0.654	52.326	92.757	107.403	151.052	349.265
Weekly mean of daily mean temperature (°C)	20.27	21.40	21.92	22.11	22.64	25.05
Weekly mean of daily maximum temperature (°C)	23.48	25.34	26.01	26.15	26.85	30.50
Weekly mean of daily minimum temperature (°C)	16.97	18.15	18.63	18.66	19.09	20.62
Weekly malaria cases – all age groups	15	128.5	199	229	313	934
Weekly malaria cases – children under 5 years	3	34	61	72.57	102.5	299
Weekly malaria cases – school-aged children (aged 5–14 years)	4	31.5	52	59.95	81	229
Weekly malaria cases – 15 years of age and above	6	56.5	83	96.73	124.5	406

### Model building and selection.

Supplemental Table 2 and Supplemental Table 3 summarize the AIC and BIC values for all model specifications tested during the first model-building step. The ERA5 daily data set yielded better AIC and BIC values in comparison with the hourly data set. The cross-basis function for rainfall was defined using a natural cubic spline, with boundary knots placed at the minimum and maximum values of the range and four equally spaced knots between the boundary knots. For temperature variables, the cross-basis function was also defined using a natural cubic spline, with boundary knots at each end of the range and three equally spaced knots between the boundary knots. The lag space was defined with knots at lags 0, 8, and 12 weeks, using 10 degrees of freedom. The stepwise model-building approach identified the model with rainfall as a single predictor as the most parsimonious and best-fitting model, based on the AIC values (12,034.99). However, considering prior knowledge that temperature is also a significant predictor of malaria incidence with a temporal lag,^13^^,^^17^^,^^38^ we chose the second-best model, which includes both rainfall and maximum temperature as predictors, as the final model. Additionally, we examined the model that included both rainfall and minimum temperature (AIC = 12,425.88) to assess whether a significant decrease in malaria risk occurred when the weekly average of daily minimum temperature dropped below 20°C, a well- established threshold in the literature.^14^^,^^39^^,^^40^

### DLNM across all age groups.

**Figure 3.**
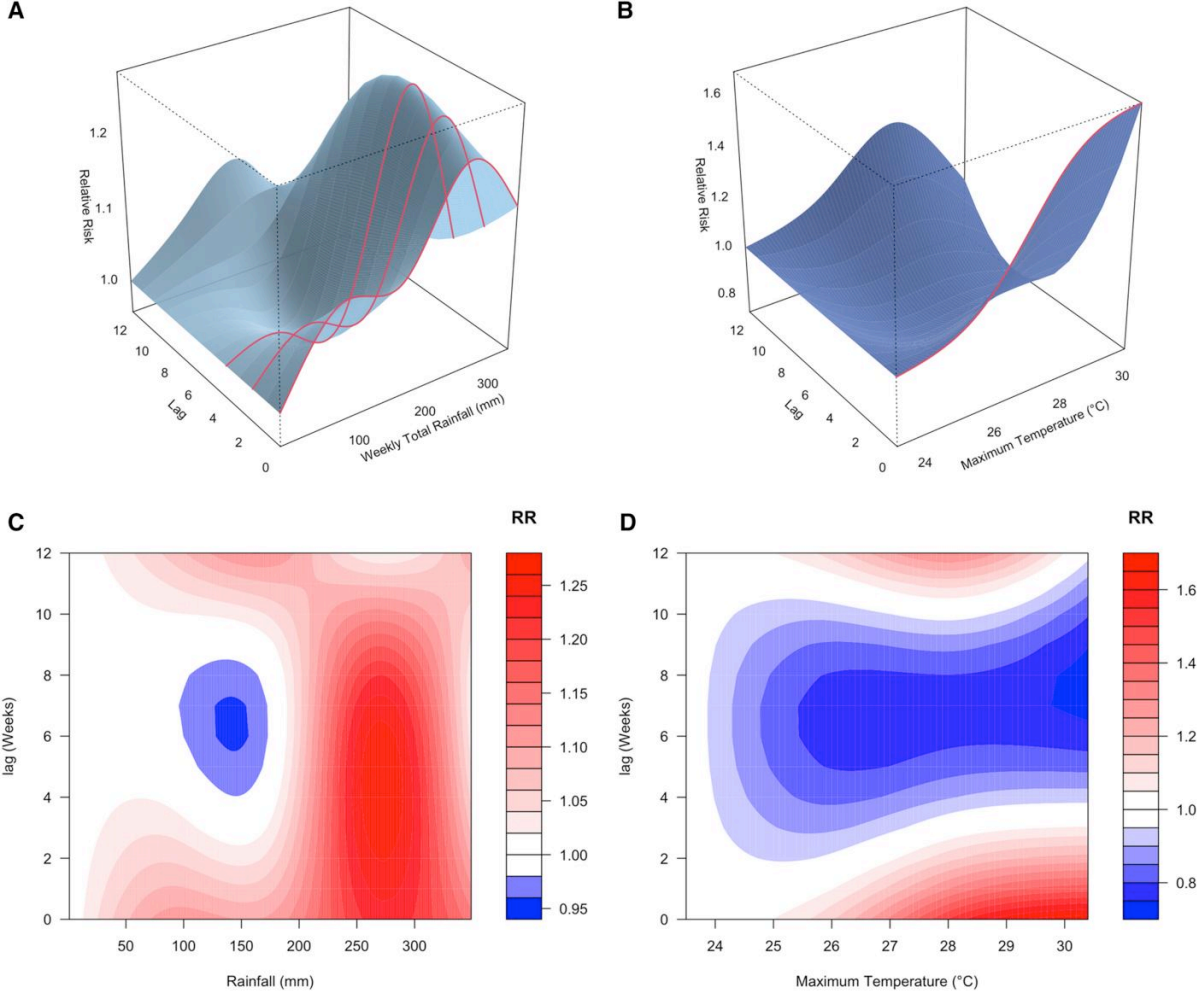
Three-dimensional (**A** and **B**) and 2-dimensional (**C** and **D**) plots summarizing the relative risk of malaria across varying rainfall (*x* axis, **A** and **C**) and temperature (*x* axis, **B** and **D**) values at different lags (*y* axis). Reference values for rainfall and maximum temperature correspond to the lowest observed values during the study period.

**Figure 4.**
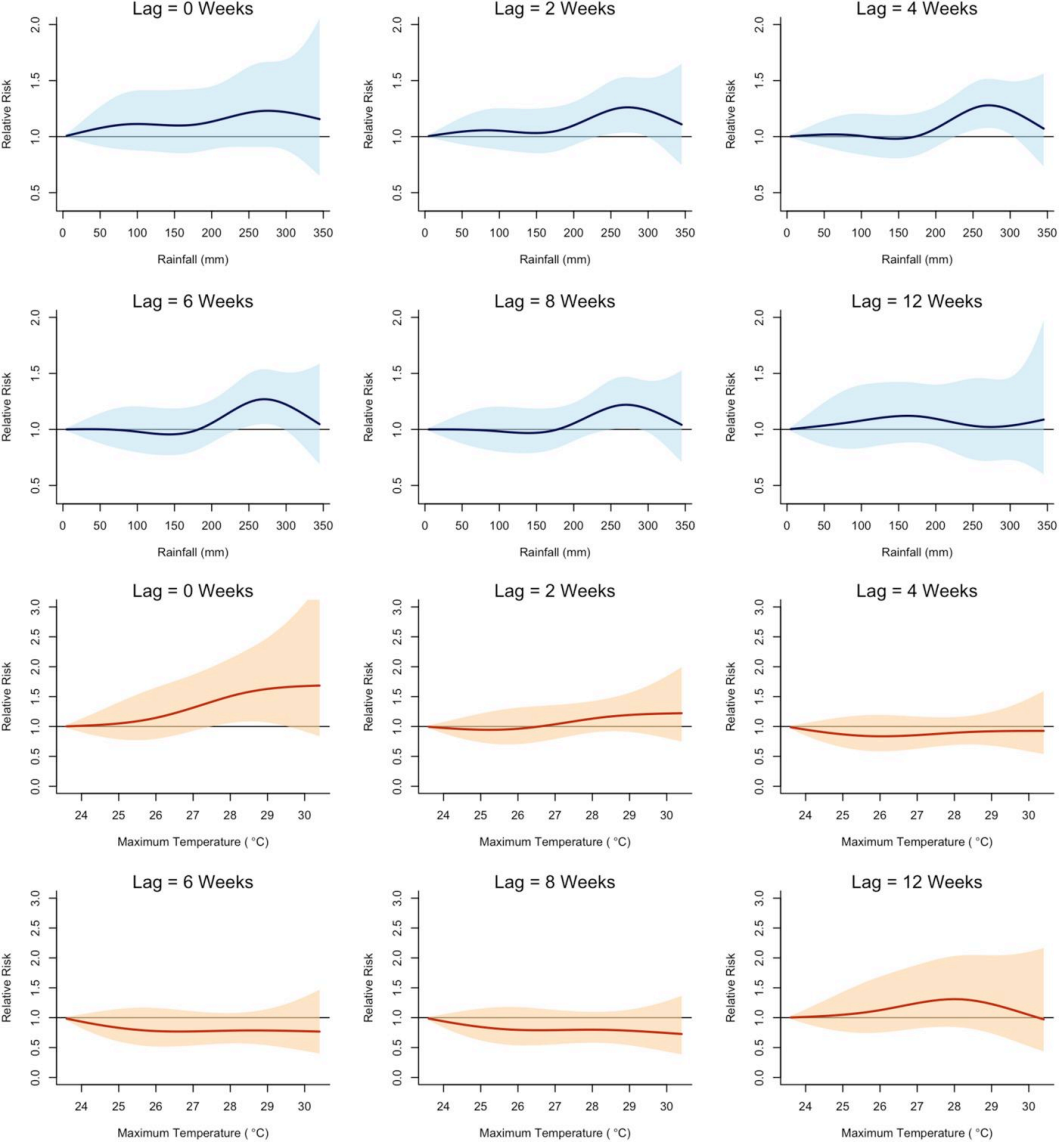
Relative risk (RR; *y* axis) of malaria across the range of observed total rainfall and maximum temperature values (*x* axis) at different lags. Reference values for rainfall and maximum temperature correspond to the lowest observed values during the study period.

**Figure 5.**
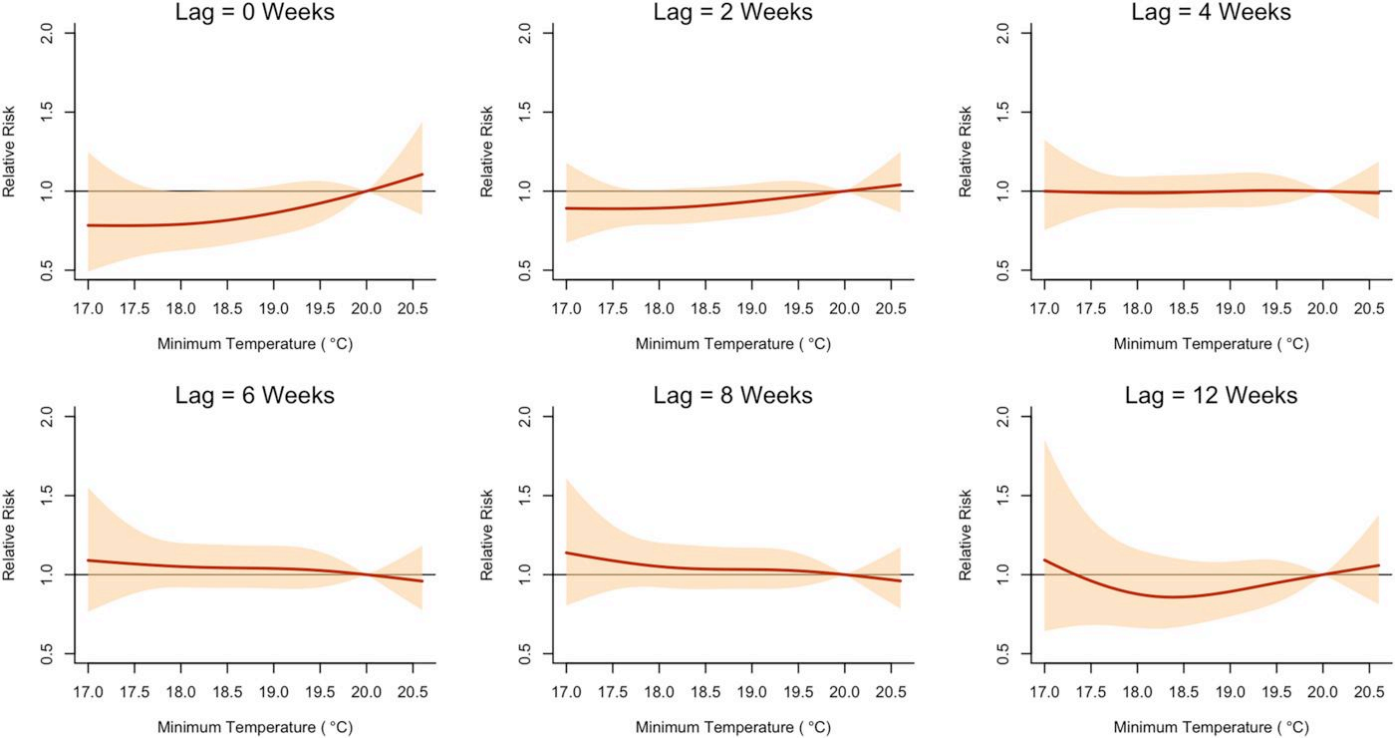
Relative risk (RR; *y* axis) of malaria across the range of observed minimum temperature values (*x* axis) at different lags. The reference value for minimum temperature is 20°C.

**Figure 6.**
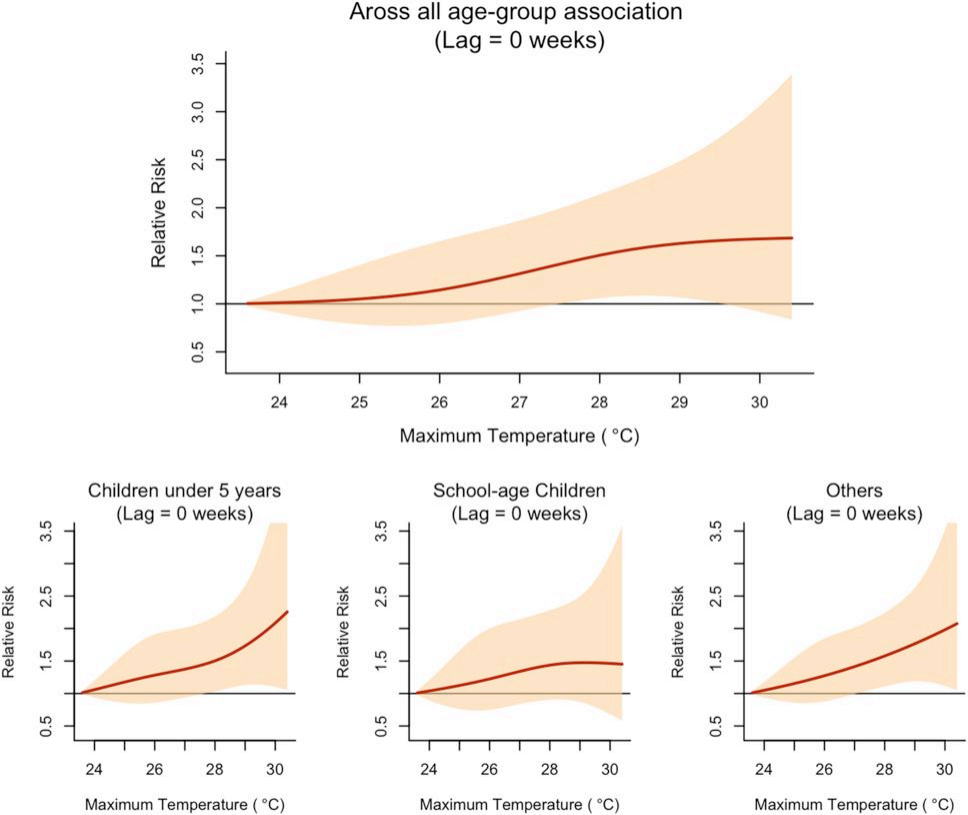
Relative risk of malaria by age group across the observed range of weekly mean daily maximum temperature (Lag = 0 weeks).

The final model (rainfall + T_max_) revealed that the relative risk (RR) of malaria began to increase at a lag of 2 weeks after exposure to approximately 220 mm of rainfall per week (Figures 3A and 3C). The RR peaked at a lag of 4 weeks, with the highest observed RR of 1.28 (95% CI: 1.08, 1.52) after exposure to a weekly total of 270 mm of rainfall (Figure 6). In contrast, the RR of malaria increased immediately (lag = 0 week) following exposure to a high daily maximum temperature of around 27.5°C (Figures 3B and 3D). The highest RR (1.65, 95% confidence interval [CI]: 1.02, 2.68) was observed immediately after exposure to a maximum daily temperature of approximately 29.4°C (Figure 4). The model including rainfall and minimum temperature did not show any significant decrease in RR when the weekly average of daily minimum temperature fell below 20°C (Figure 5).

### Age group–specific analysis.

**Figure 7.**
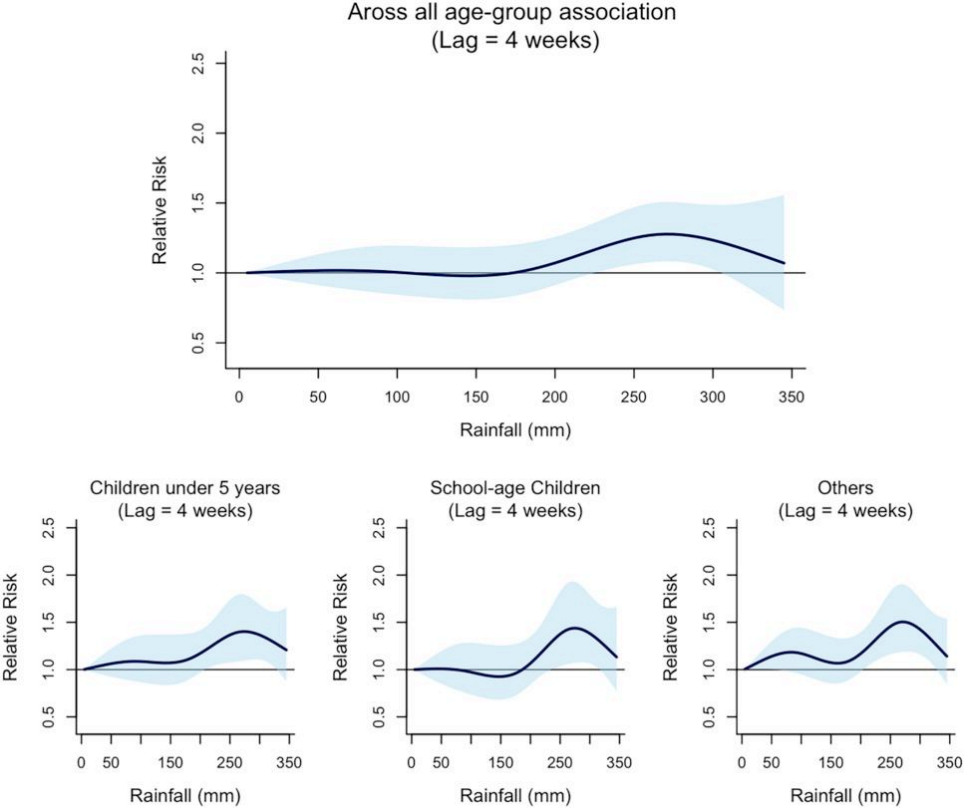
Relative risk of malaria by age group across the observed range of weekly total rainfall (Lag = 4 weeks).

DNLM analyses repeated for specific age groups showed that the risk of malaria among school-aged children was less sensitive to climate variables in comparison with the other two age groups. Figures 6 and 8 show age-specific RRs across the observed range of weekly mean daily maximum temperature and weekly total rainfall, respectively. Both children under 5 years of age and individuals aged 15 years or older were at significantly higher risk of malaria when the weekly average of daily maximum temperature exceeded the threshold. However, the RR among school-age children did not significantly increase under the same high-temperature exposure. At a 4-week lag, children under 5 years and individuals aged 15 years or older were at significantly higher risk of malaria when exposed to a total weekly rainfall exceeding 200 mm (Figure 7). In contrast, for school-aged children, this threshold was higher, with a significantly increased risk of malaria only when total weekly rainfall exceeded 250 mm.

## DISCUSSION

This study marks the first attempt to use the longitudinal malaria case data from the Iganga-Mayuge HDSS site to quantify the exposure-lag-response relationships between climate variables and malaria risk, using a DLNM approach. This methodology allowed us to investigate the delayed and nonlinear effects of climate variables on malaria in a flexible manner. Our analysis revealed that malaria risk increases significantly after periods of heavy rain. Specifically, we found that when weekly rainfall exceeds 200 mm, the risk of malaria increases after 2 weeks and can remain high up to 8 weeks. The highest increase in risk occurred 4 weeks after weekly rainfall reached 270 mm, at which point the risk was 28% higher than normal. In high-burden settings like Eastern Uganda, this level of increased risk could lead to a significant surge in malaria cases and place added strain on health care resources. The 2- to 8-week lag aligns with the expected mosquito life cycle and the parasite development within the mosquito. Heavy rainfall typically creates standing water, which provides ideal breeding grounds for mosquitoes. The 4- to 8-week lag is consistent with the time it takes for mosquitoes to lay eggs and for the eggs to develop into larvae, pupae, and then adult mosquitoes that are capable of transmitting malaria,^41^^,^^42^ the typical incubation period for the malaria parasite to develop within the mosquito,^41^ as well as the time it takes for the malaria parasite to develop within the human host,^35^^,^^41^ for the onset of malaria symptoms, and for individuals to seek health care. A study conducted in Zambia found that sustained humidity could increase malaria risk by facilitating more foraging by resting adult mosquitoes,^43^ and this increased foraging could explain the observed increased malaria risk at 2 weeks in this study.

Conversely, our analysis did not reveal any significant lagged association between temperature and malaria risk. Although we noted an immediate increase in malaria cases (lag = 0 weeks) when daily maximum temperatures ranged from 27.5°C to 29.5°C, this association did not persist over longer lag periods. Further, contrary to established knowledge that malaria risk decreases when temperatures drop below 20°C,^14^^,^^39^^,^^40^ we observed no significant decrease in malaria risk associated with daily minimum temperatures. This finding may be because our study site lies within the Lake Victoria climate zone, which experiences minimal seasonal variation in temperature.^44^^,^^45^

The age-stratified analysis revealed that school-aged children showed a lower sensitivity to climate in terms of malaria risk in comparison with other age groups. We believe this finding may stem from our malaria data being sourced from a single facility within the study area, which captures only symptomatic cases of individuals who sought medical care at this facility. Although school-aged children report the highest prevalence of parasitemia,^34^ most infections in this group are asymptomatic or, when symptomatic, tend to be less severe.^34^^,^^36^ Consequently, these circumstances could lead to underreporting of malaria cases among school-aged children at health facilities, because they may not seek care when experiencing mild symptoms.^46^ One possible reason for this underreporting could be the targeted malaria prevention interventions in Ugandan schools, such as the distribution of long-lasting insecticide treated nets and education campaigns,^47^ along with school-based malaria chemoprevention.^48^ These efforts may have increased awareness and compliance with preventive measures in this age group in comparison with others.^47^ However, we were unable to find concrete evidence of such interventions in the study area. Another explanation is that school-aged children may engage in behaviors and activity patterns that differ from those of other age groups, which could mitigate their exposure to the heightened malaria risk associated with climatic factors.

Our findings offer insights to optimize the timing of malaria preventive interventions and strengthening preparedness to address increased demand for treatment. In our study setting, rainfall emerged as the most significant predictor of elevated malaria risk. The observed lag period of 2 to 8 weeks between heavy rainfall and increased malaria risk presents a critical window for intervention. Given the malaria parasite’s life cycle, rainfall exceeding 200 mm per week could serve as an early warning for implementing interventions. Public health authorities could operationalize this lead time by ramping up vector control measures, including distributing bed nets and conducting indoor residual spraying, as well as engaging communities with educational campaigns on malaria awareness and prevention and preparing health facilities with adequate supplies of diagnostic tests and antimalarials. This proactive approach can help mitigate the anticipated surge in cases and reduce the overall disease burden. However, the feasibility and effectiveness of public health response will largely depend on the operational capacity of the health system, the costs and effectiveness of available interventions, the local implementation context, and the availability of resources. Furthermore, the benefits of acting on early warnings must outweigh its costs, particularly given the inherent uncertainties in climate-based disease forecasting.^49^ Notably, the increased malaria risk was particularly pronounced in children under 5 years and individuals aged 15 years and older. Promoting the uptake of intermittent preventive treatment among pregnant women could be an effective way to also mitigate malaria cases in young children. Moreover, future studies should investigate the relationship between climate variables and malaria infection among school children, especially asymptomatic parasitemia. This exploration will enhance our understanding of this age group’s role in sustaining malaria transmission and equip local health authorities with crucial insights to develop targeted intervention strategies for this age group.

Our study has several limitations. First, the remotely sensed climate datasets used in our analysis may not accurately represent ground-level temperature and rainfall measurements. Discrepancies between satellite observations and on-the-ground data can result from various factors, including smoothing errors, measurement inaccuracies, and *a priori* assumptions.^50^ To mitigate this issue, we compared climate datasets from three distinct sources to identify the most comprehensive and reliable option. However, we recommend supplementing satellite data with direct measurements from ground-level weather stations whenever feasible. Second, we relied on malaria case data from a single health facility within the HDSS site, which likely underrepresents the total malaria burden in the study area and may introduce bias. This dataset captured only symptomatic people who sought care, excluding individuals who were unable to access care due to distance, economic constraints, or other barriers, as well as individuals with asymptomatic infections or those who sought treatment at different health facilities. Further, the DLNM approach is known for its complexity, requiring numerous model specifications and exhibiting sensitivity to various model parameters, which can complicate model selection.^30^ Our model specifications and selections were informed by our knowledge of the study area, our prior experience with DLNM models to other climate–disease associations,^51^^–^^54^ and suggestions from experts.^30^^,^^37^ Nonetheless, we acknowledge that variations in model specifications and parameters, depending on the model builder, can lead to different results because of these inherent complexities. Last, this study did not include external validation of the final model due to data constraints. Future research should assess the generalizability of our findings using independent datasets from other settings or time periods.

## CONCLUSION

In conclusion, our study reveals a nuanced relationship between local climate and malaria risk, providing critical insights for targeted public health interventions. The identified lag periods offer a strategic window for deploying preventive measures, such as bed-net distributions and education campaigns, based on heavy rainfall forecasts. Our age group–specific analysis highlights the unique resilience of school-aged children, likely influenced by distinct daily activity patterns, a higher likelihood of asymptomatic infections due to stronger immunity from early childhood exposure, and targeted interventions for this subgroup.

Given the study’s limitations, we emphasize the need for ongoing research to refine methodologies, incorporate ground-level climate data, conduct external validation using independent datasets, and explore the multifaceted nature of malaria transmission dynamics. Our findings contribute to the existing evidence on climate–malaria associations, while fostering a deeper understanding of the exposure-lag-response relationships, which can inform malaria interventions in our study setting. Our findings also underscore the complexity of climate–malaria interactions and advocate for a comprehensive and context-specific approach to malaria prevention and control in endemic settings. This research aligns with global efforts to mitigate the burden of malaria, particularly in vulnerable populations, and promotes preparedness and resilience in vector-borne disease control in the face of a rapidly changing climate.

## Supplemental Materials

10.4269/ajtmh.25-0031 Supplemental Materials
