# Supplementary material for: Quantifying the Lagged Effects of Climate Variables on Malaria Risk in Eastern Uganda
Source: Am J Trop Med Hyg. 2025 Oct 7;113(6):1256–66. doi: 10.4269/ajtmh.25-0031 (PMC12676583; doi:10.4269/ajtmh.25-0031)
Supplement: Supplemental Materials [file tpmd250031.SD1.pdf]

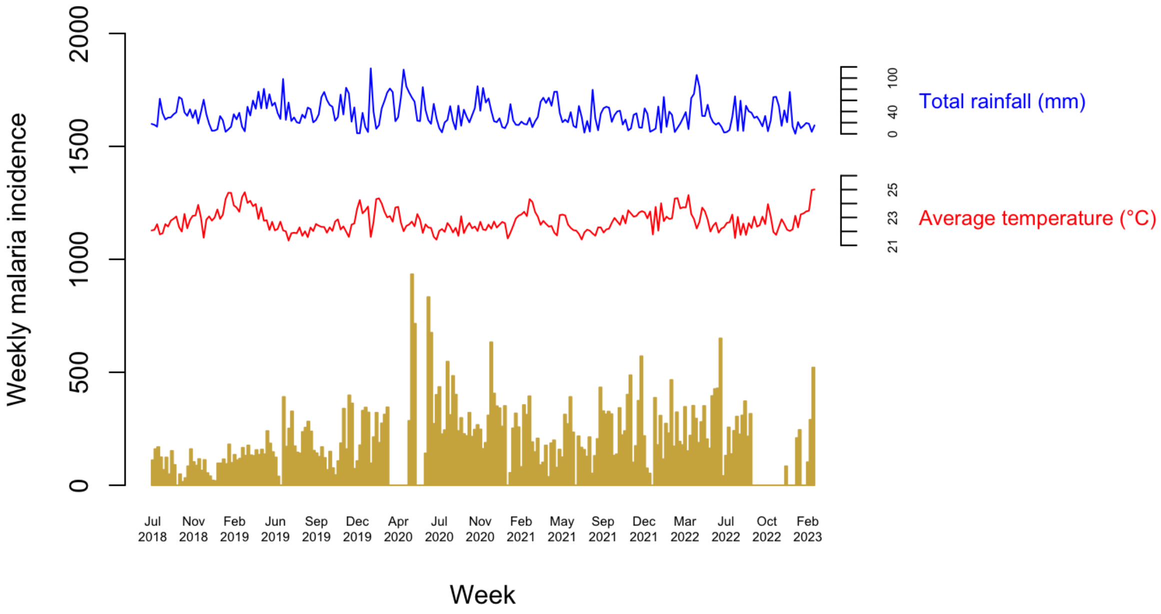

Weekly malaria incidence
0
500
1000
1500
2000
Total rainfall (mm)
0
40
100
Average temperature (°C)
21
23
25
Jul 2018
Nov 2018
Feb 2019
Jun 2019
Sep 2019
Dec 2019
Apr 2020
Jul 2020
Nov 2020
Feb 2021
May 2021
Sep 2021
Dec 2021
Mar 2022
Jul 2022
Oct 2022
Feb 2023
Week

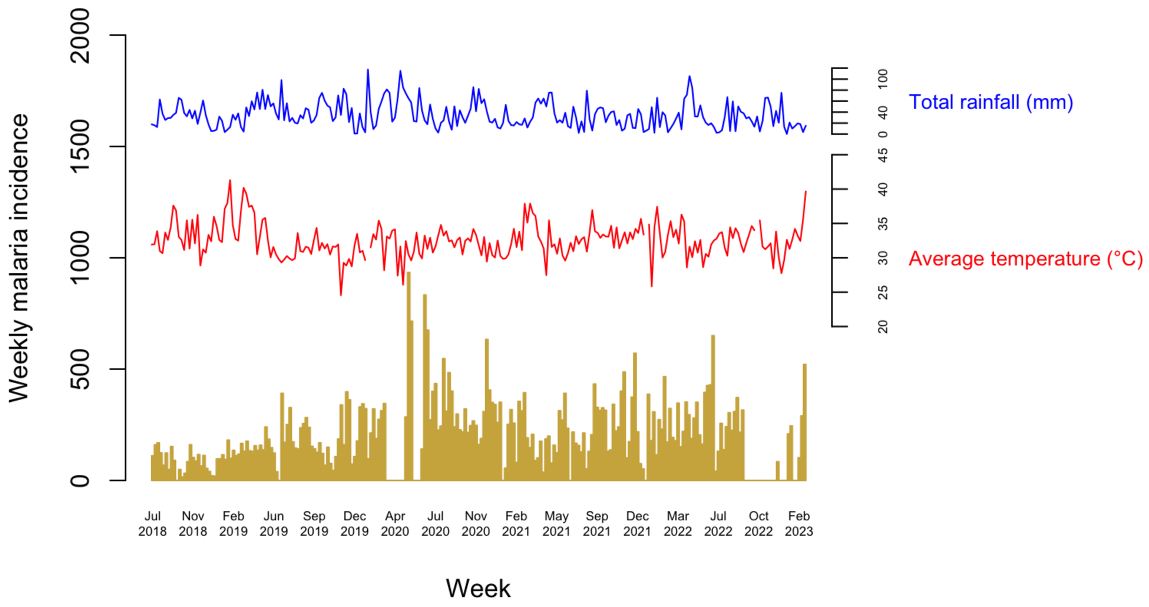

Weekly malaria incidence
2000
1500
1000
500
0
Total rainfall (mm)
Average temperature (°C)
Jul 2018
Nov 2018
Feb 2019
Jun 2019
Sep 2019
Dec 2019
Apr 2020
Jul 2020
Nov 2020
Feb 2021
May 2021
Sep 2021
Dec 2021
Mar 2022
Jul 2022
Oct 2022
Feb 2023
Week

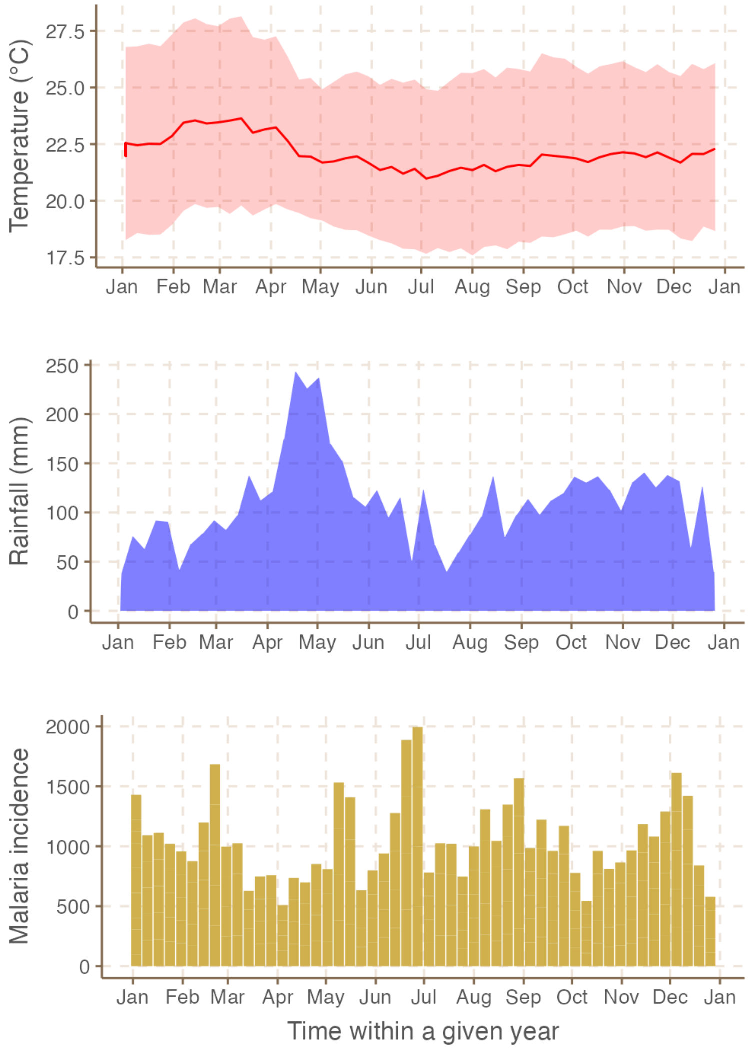

Temperature (°C)
27.5
25.0
22.5
20.0
17.5
Jan Feb Mar Apr May Jun Jul Aug Sep Oct Nov Dec Jan
Rainfall (mm)
250
200
150
100
50
0
Jan Feb Mar Apr May Jun Jul Aug Sep Oct Nov Dec Jan
Malaria incidence
2000
1500
1000
500
0
Jan Feb Mar Apr May Jun Jul Aug Sep Oct Nov Dec Jan
Time within a given year

S1 Table. Different specifications of the distributed lag non-linear model (DLNM) that were tested in the study.

Model aspects	Tested options
Climate data set	Complete ERA5 global atmospheric reanalysis with a spatial resolution of 0.25° X 0.25° [28];<br>ERA5- ERA5-Land daily aggregated -EMCWF climate reanalysis with a spatial resolution of 0.1° X 0.1° [29];<br>MOD21A1D.061 Terra Land Surface Temperature and 3-Band Emissivity Daily Global from NASA LP DAAC with a spatial resolution of 1km X 1km [30]
Spline function shape	Natural cubic spline<br>B-spline with 2 degrees of freedom<br>B-spline with 3 degrees of freedom
Variable knot	Equally spaced 4 knots<br>Equally spaced 3 knots<br>Quantile knots<br>Other knots determined based on eyeball observation of the variable distribution
Lag knot (weeks)	[2,4,6,8,10]<br>[2,4,8]<br>[4,8]<br>[2,8]<br>[8]
Time degree of freedom	10, 20, 40
Model parameters	Weekly mean of average daily temperature (Temp, °C)<br>Weekly mean of minimum daily temperature ($T_{min,}$ °C)<br>Weekly mean of maximum daily temperature (T_{max}, °C)<br>Weekly total rainfall (rain, mm)

S2 Table. Model performance evaluation using different model specifications

Cross-basis function for rainfall variable		Cross-basis function for temperature variable		Smoothed time function		ERA Complete analysis (hourly)		ERA daily - temp	
Spline	Knots	Spline	Knots	Lag knots	df*	AIC**	BIC***	AIC	BIC
Natural cubic spline	3 knots at a lower quartile, median, and upper quartile point	Natural cubic spline	3 knots at a lower quartile, median, and upper quartile point	5 knots at weeks 2, 4, 6, 8, and 10	20	14178.45	7547.526	13923.08	7439.564
Natural cubic spline	4 knots at 17, 30, 60, 90	Natural cubic spline	3 knots at a lower quartile, median, and upper quartile point	5 knots at weeks 2, 4, 6, 8, and 10	20	14415.99	7215.961	12081.13	6214.888
Natural cubic spline	4 knots at 17, 30, 60, 90	Natural cubic spline	3 knots at a lower quartile, median, and upper quartile point	5 knots at weeks 2, 4, 6, 8, and 10	10	13991.15	7628.894	12068.06	6740.231
Natural cubic spline	4 knots at 17, 30, 60, 90	B-spline (df = 3)	3 knots at a lower quartile, median, and upper quartile point	5 knots at weeks 2, 4, 6, 8, and 10	20	13444.54	6178.094	11779.25	5498.103
Natural cubic spline	4 knots at 17, 30, 60, 90	B-spline (df = 3)	3 knots at a lower quartile, median, and upper quartile point	5 knots at weeks 2, 4, 6, 8, and 10	10	13490.12	6668.942	11954.79	6036.663
B-spline (df = 3)	4 knots at 17, 30, 60, 90	Natural cubic spline	3 knots at a lower quartile, median, and upper quartile point	5 knots at weeks 2, 4, 6, 8, and 10	20	13224.49	5998/568	12283.19	5664.18
B-spline (df = 3)	4 knots at 17, 30, 60, 90	B-spline (df = 3)	3 knots at a lower quartile, median, and upper quartile point	5 knots at weeks 2, 4, 6, 8, and 10	20	12635.48	5269.221	11793.62	4985.235
B-spline (df = 3)	4 knots at 17, 30, 60, 90	B-spline (df = 3)	3 knots at a lower quartile, median, and upper quartile point	5 knots at weeks 2, 4, 6, 8, and 10	10	12724.59	5702.45	12702.7	5636.211
B-spline (df = 3)	4 knots at 17, 30, 60, 90	B-spline (df = 2)	3 knots at a lower quartile, median, and upper quartile point	5 knots at weeks 2, 4, 6, 8, and 10	20	11309.02	5146.839	12258.57	5369.048
Natural cubic spline	4 equally-spaced knots	Natural cubic spline	4 equally-spaced knots	5 knots at weeks 2, 4, 6, 8, and 10	10	13415.06	7827.247	13024.85	7630.969
Natural cubic spline	4 equally-spaced knots	Natural cubic spline	3 equally-spaced knots	5 knots at weeks 2, 4, 6, 8, and 10	10	13166.62	8188.306	13463.53	8309.207
Natural cubic spline	4 equally-spaced knots	B-spline (df = 2)	3 equally-spaced knots	5 knots at weeks 2, 4, 6, 8, and 10	10	13414.98	7819.642	12730.69	7488.159
Natural cubic spline	4 equally-spaced knots	B-spline (df = 2)	4 equally-spaced knots	5 knots at weeks 2, 4, 6, 8, and 10	10	13735.33	7541.58	12970.99	7201.374
Natural cubic spline	4 equally-spaced knots	Natural cubic spline	First 2 of the 4 equally-spaced knots	5 knots at weeks 2, 4, 6, 8, and 10	10	13246.77	8220.83	13552.8	8375.432
Natural cubic spline	4 equally-spaced knots	B-spline (df = 2)	4 equally-spaced knots	5 knots at weeks 2, 4, 6, 8, and 10	20	14165.12	7141.188	12958.27	6601.144
Natural cubic spline	4 equally-spaced knots	Natural cubic spline	4 equally-spaced knots	5 knots at weeks 2, 4, 6, 8, and 10	40	13866.52	6299.673	13316.07	6114.242
Natural cubic spline	4 equally-spaced knots	Natural cubic spline	3 equally-spaced knots	1 knot at week 8	10			12247.27	9537.813
Natural cubic spline	4 equally-spaced knots	Natural cubic spline	First 2 of the 4 equally-spaced knots	1 knot at week 8	10			12294.75	9564.771

Natural cubic spline	4 equally-spaced knots	Natural cubic spline	First 2 of the 4 equally-spaced knots	2 knots at weeks 2 and 8	10			12737.1	9328.98
Natural cubic spline	4 equally-spaced knots	Natural cubic spline	First 2 of the 4 equally-spaced knots	2 knots at weeks 4 and 8	10			12616.16	9242.074
Natural cubic spline	4 equally-spaced knots	Natural cubic spline	equal space knot (4) - first two	3 knots at weeks 2, 4 and 8	10			12519.44	8687.107

*df = Degree of Freedom; **AIC = Aikake Information Criteria; ***BIC = Bayesian Information Criteria

S3 Table. Model performance evaluation results for the forward stepwise model building process *

Model	AIC	BIC
T_{min}	12300.73	10467.66
T_{max}	12995.57	11040.35
Temp	12377.69	10524.54
Rain	12034.99	10027.65
T_{min} + rain	12425.88	9649.099
T_{max} + rain	12217.92	9520.322
temp + rain	12247.27	9537.813
T_{min} + T_{max} + rain	12392.86	9019.85
T_{min} + temp + rain	12767.27	9244.078
T_{max} + temp + rain	12261.37	8913.159
T_{min} + T_{max} + temp + rain	12501.77	8428.429
*Note: All of the models used the cross-basis function for rainfall using a natural cubic spline with two terminal knots and four equally spaced knots in between, the cross-basis function for temperature using a natural cubic spline with two terminal knots and three equally-spaced knots, and a smoothed time function with knots at 0, 8, and 12 weeks and 10 degrees of freedom.		
